# The Roles of SNF2/SWI2 Nucleosome Remodeling Enzymes in Blood Cell Differentiation and Leukemia

**DOI:** 10.1155/2015/347571

**Published:** 2015-02-19

**Authors:** Punit Prasad, Andreas Lennartsson, Karl Ekwall

**Affiliations:** Department of Biosciences and Nutrition, Karolinska Institute, 141 57 Huddinge, Sweden

## Abstract

Here, we review the role of sucrose nonfermenting (SNF2) family enzymes in blood cell development. The SNF2 family comprises helicase-like ATPases, originally discovered in yeast, that can remodel chromatin by changing chromatin structure and composition. The human genome encodes 30 different SNF2 enzymes. SNF2 family enzymes are often part of multisubunit chromatin remodeling complexes (CRCs), which consist of noncatalytic/auxiliary subunit along with the ATPase subunit. However, blood cells express a limited set of SNF2 ATPases that are necessary to maintain the pool of hematopoietic stem cells (HSCs) and drive normal blood cell development and differentiation. The composition of CRCs can be altered by the association of specific auxiliary subunits. Several auxiliary CRC subunits have specific functions in hematopoiesis. Aberrant expressions of SNF2 ATPases and/or auxiliary CRC subunit(s) are often observed in hematological malignancies. Using large-scale data from the International Cancer Genome Consortium (ICGC) we observed frequent mutations in genes encoding SNF2 helicase-like enzymes and auxiliary CRC subunits in leukemia. Hence, orderly function of SNF2 family enzymes is crucial for the execution of normal blood cell developmental program, and defects in chromatin remodeling caused by mutations or aberrant expression of these proteins may contribute to leukemogenesis.

## 1. Introduction

The gene encoding the first SNF2/SWI2 enzyme was discovered by the yeast geneticists Ira Herskowitz and Marian Carlson in the 1980s. These researchers named the gene two different names depending on the genetic screen used for their identification [1, 2]: sucrose nonfermenting mutant (*SNF2*) and mating-type switching mutant (*SWI2*). Analysis of chromatin from these mutants* in vivo* suggested that the gene products affected chromatin structure [3]. Approximately 10 years after their genetic discovery, the yeast SWI/SNF protein complex was purified. It was demonstrated to remodel nucleosomes* in vitro* and to affect the binding of the transcription factor GAL4 [4]. The yeast community now uses the* SNF2* gene name (http://www.yeastgenome.org/), and we use this nomenclature in this review article.

**Figure 1:**
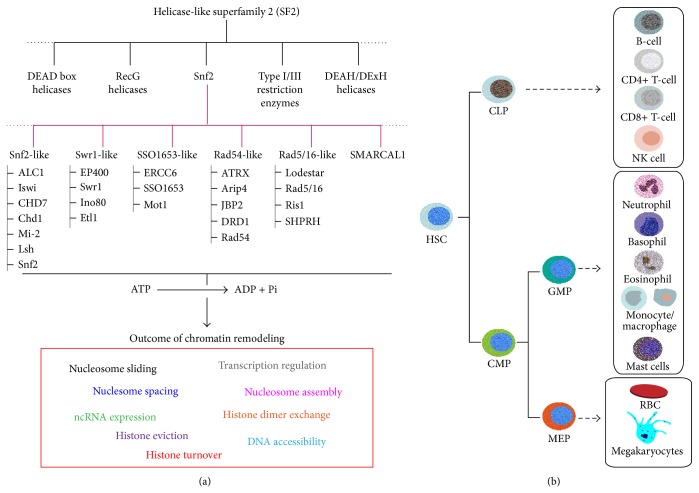
Classification of SNF2 enzymes and schema of mammalian hematopoiesis. SNF2-like chromatin remodelers belong to SF2 superfamily and are classified based on conserved structure of the ATPase domain as discussed in Flaus et al. [5] (a). Overview of mammalian hematopoietic cell development and differentiation. Dashed arrows show intermediate stages of hematopoietic development which is not shown in the figure for simplicity purpose (b).

A SNF2 protein is an enzyme that belongs to the SF2 helicase-like superfamily, and it is the founding member of a subfamily of enzymes called SNF2-like helicases, which all harbor a conserved helicase-related motifs similar to SNF2 [5]. The SNF2 family proteins have multiple members, which are approximately 30 different enzymes in human cells (53 different enzymes, including all the splice variants) and 17 different enzymes in budding yeast. SNF2 enzymes can be further classified into six groups based on the structure of the helicase domain. These groups are Swi2/Snf2-like, Swr1-like, SS01653-like, Rad54-like, Rad5/6-like, and distant (SMARCAL1) enzymes (Figure 1(a)) [5]. Many of the SNF2 enzymes have been shown to remodel chromatin* in vitro* in an ATP-dependent manner, and several enzymes remain to be tested.

Because SNF2 enzymes regulate DNA accessibility in chromatin fibers, they are important regulators of gene expression and genome stability. SNF2 enzymes are key players in epigenetic control. They affect several epigenetic modification processes, including DNA methylation, histone modification, histone variant exchange, noncoding RNA, and higher order chromatin structure [6]. SNF2 enzymes also function downstream of epigenetic modifications targeted to acetylated chromatin via a special domains to remodel chromatin. For example, the SNF2 enzyme SMARCA4/Brg1 is targeted via a Bromodomain [6, 7].

Numerous* in vivo* and biochemical studies have been employed using different model organisms to address the detailed effects of SNF2 enzymes on chromatin. The chromatin remodeling reaction can lead to nucleosome sliding, histone exchange, histone eviction (disassembly), and nucleosome spacing to form regular arrays or nucleosome assembly depending on both which SNF2 enzyme is used and whether other cofactors, such as histone chaperones, are added to the experiments (recently reviewed in [6]). The disassembly function is particularly important in gene regulation to ensure that promoter and enhancer DNA sequences are accessible for transcription factors, epigenetic modifiers, and RNA polymerase II. SNF2 enzymes are often part of multisubunit chromatin remodeling complexes (CRC) containing several auxiliary subunits.

## 2. Chromatin Reorganization during Hematopoiesis

The hematopoietic system consists of two main cell lineages, the myeloid and the lymphoid, which both originate from hematopoietic stem cells (HSCs) (Figure 1(b)). Briefly, multipotent HSCs differentiate to give rise to common myeloid progenitors (CMPs) and common lymphoid progenitors (CLPs). Further differentiation of CMPs yields megakaryocyte/erythroid progenitor (MEP) and granulocyte-monocyte progenitor (GMP). The MEP differentiates and matures into erythrocytes and megakaryocytes, whereas the GMP differentiates into monocytes and granulocytes (neutrophils, eosinophils, and basophils), which are the first line of defense against infections [8]. CLPs give rise to T-cells, B-cells, and Natural Killer (NK) cells. The lymphoid subtypes of hematopoietic cells are crucial for adaptive immunity. Final B-, T-, and NK-cell maturation occur in the peripheral lymphoid tissue, where antigen selection takes place.

Lineage choice and blood cell differentiation are tightly regulated at several levels. Different transcription factors (TFs) have been identified that together form a regulatory network for hematopoiesis (reviewed in [9]). Several TFs have been shown to interact with epigenetic regulators, including CRCs such as the Nucleosome Remodeling and deacetylase (NuRD) complex (reviewed in [8]). The importance of chromatin remodelers in hematopoiesis was recently suggested by two studies, which demonstrated a massive chromatin reorganization during blood cell differentiation [10, 11]. Both studies elegantly showed how chromatin compaction is modified in enhancer regions during hematopoiesis in a lineage-specific manner [10, 12]. H3K4 monomethylation and chromatin decompaction were demonstrated to precede H3K27 acetylation and enhancer activation [10]. Thus, nucleosome reorganization is highly regulated in a stepwise manner that likely includes both transcription factors and CRCs. Additionally, human nucleosome organization differs between mature cell types from myeloid and lymphoid lineages [11]. Detailed analysis of nucleosome positioning with micrococcal nuclease (MNase) combined with parallel sequencing clearly showed significant cell type-specific differences in nucleosome organization between granulocytes and T-cells [11]. The linker length between nucleosomes in granulocytes is 46 base pairs but 56 base pairs in T-cells [11]. Differences in chromatin organization are not surprising given the polylobular nuclear shape in granulocytes that differs dramatically from that of the small round nuclei in T-cells. Because all blood cell types stem from a common HSC, a major nucleosome reorganization must occur during hematopoiesis to yield all the various blood cell types. Thus, SNF2 family enzymes and associated auxiliary subunits play important roles in blood cell differentiation.

## 3. Specific Roles for SNF2 Family Enzymes in Hematopoietic Cell Differentiation

How alterations in chromatin structure allow for temporal control of gene expression to orchestrate the different developmental programs is just now beginning to unfold. A recent study in Zebrafish by Huang et al. found several chromatin modulators, including CRCs such as BAF, ISWI, and NuRD, to be key regulators of hematopoiesis [13]. Here, we review how the various hematopoietic lineages are regulated by a network of TFs and associated ATP-dependent chromatin remodeling factors.

### 3.1. SWI/SNF

The SWI/SNF complex has been extensively studied in model organisms, where it both activates and represses gene expression [14]. In mammals, the approximately 2 MDa-sized SWI/SNF complex exists with two distinct catalytic cores, either Brahma (BRM)/SMARCA2 or the product of the Brm related gene1 (BRG1)/SMARCA4. The BRG/BRM complexes contain both common and distinct auxiliary factors and have therefore been named BAF complexes (containing BRG/BRM associated factors) [15]. Mouse genetics has been instrumental to elucidating SWI/SNF functions in development. Another subcomplex of BAF, called PBAF (polybromo associated factors), consists of a SMARCA4 catalytic core along with BAF200/ARID2 and BAF180/PBRM1 auxiliary factors. Biochemically both BAF and PBAF complex have similar nucleosome remodeling activity [16].* Brg1* ^*−/−*^ mouse embryos die at the preimplantation stage, whereas* Brm* ^*−/−*^ mice develop normally [17]. This pioneering study by Reyes et al. also demonstrated that SMARCA4 expression is enhanced in the absence of SMARCA2, suggesting that SMARCA2 is dispensable and that SMARCA4 has a role that is essential for early embryonic development. Since their study, further research has revealed the precise functions of BAF complexes in mouse embryogenesis, organogenesis, and tissue formation [18, 19].

**Figure 2:**
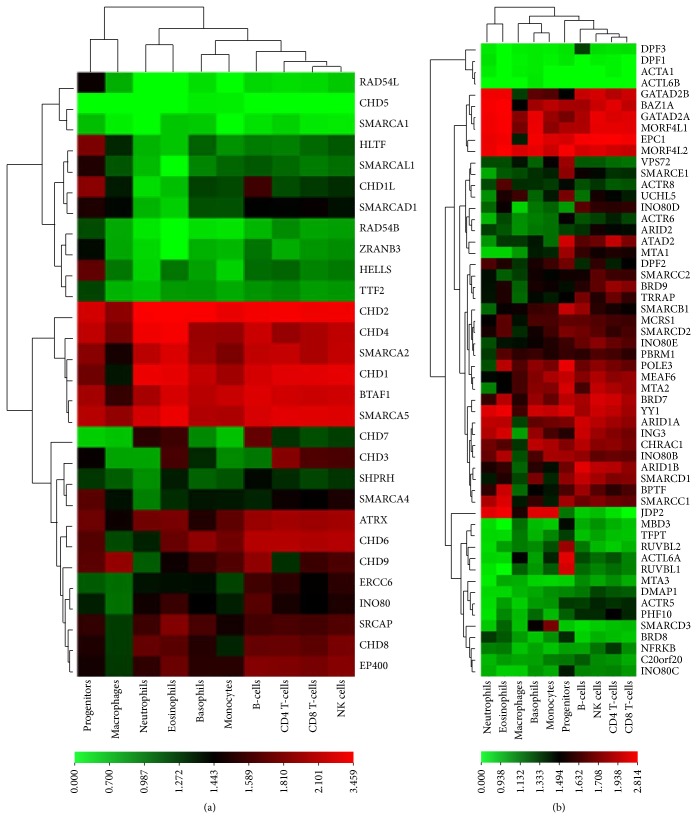
Expression of human CRCs and their cofactors in normal hematopoietic cells. The CAGE expression data were extracted from [28], and tags per million values were increased by a unit and converted to log10 values. The hierarchal clustering and heatmaps were constructed using one matrix clustered image maps (CIMminer) using the Euclidean distance method (http://discover.nci.nih.gov/cimminer/home.do) for the CRC ATPase subunit (a) and associated auxiliary cofactors (b).

BAF complexes are implicated in HSC maintenance [20] and regulation of erythroid [21], lymphoid [22], and myeloid lineages [23]. The SMARCA4 complex contains several BAF subunits. These subunits play essential roles in maintaining the integrity of the complex and support the function of the complex [24]. For example, mice bearing the* Baf155* ^*−/−*^ mutation die at the preimplantation stage, similar to* Brg1* ^*−/−*^ null mutation [25]. Mice heterozygous for a mutation in* Baf155* ^*−*/+^ show defects in brain organization [25], whereas* Baf155* ^*−/−*^ homozygous mutant mice harboring a transgene expressing BAF155/SMARCC1 show a defect in blood vessel formation [26]. These findings show an important function for the auxiliary subunit SMARCC1 in neurogenesis and illustrate the importance of having a correct gene dosage for hematopoietic physiology. A mutation in SMARCB1, another integral component of the BAF complex, results in bone marrow failure and T-cell lymphoma [27]. We recently showed that ACTL6A displays hematopoietic progenitor-specific expression (Figure 2(b)) [28]. Krasteva et al. recently demonstrated with functions in HSC maintenance and proliferation [20].

In the erythroid lineage, a SMARCA4-containing BAF complex is essential for both primitive and definitive erythropoiesis [29, 30]. Erythropoiesis depends on the efficient recruitment and chromatin remodeling by SMARCA4 to enable the transcription of the embryonic and adult *β*-globin genes. This mechanism was discovered by Bultman et al. using a hypomorph point mutation (E1083G) located in the ATPase domain of SMARCA4. This mutation does not affect BAF complex integrity, ATP hydrolysis, or recruitment of the complex to the adult *β*-globin genes. However, this mutation does compromise the chromatin remodeling activity of SMARCA4 and leads to a closed chromatin confirmation, thereby inhibiting the transcription of *β*-globin genes for definitive erythropoiesis [29]. However, a* Brg1* ^*−/−*^ conditional null mutation affects the expression of the *α*-globin locus and, hence, primitive erythropoiesis [30]. This finding suggests that the E1083G mutation may have a locus-specific chromatin remodeling activity that is critical only for *β*-globin gene expression.

The T-cells that migrate to thymocytes from bone marrow do not possess CD4 or CD8 antigen coreceptors and are thus described as double negative (DN) cells. During subsequent stages of T-cell differentiation in the thymus, the CD8 receptor is activated, followed by depression of the CD4 marker or vice versa. Once the T-cells become CD4+ or CD8+, they undergo a final maturation [31]. The BRG1 complex is crucial for T-cell development, CD4+ CD8− (helper T-cells)/CD4− CD8+ (cytotoxic T-cells) lineage choice and T-cell activation [22, 31, 32]. One of the earliest studies by Zhao et al. demonstrated that BRG1 and its associated auxiliary cofactor BAF53 are required for the dynamic chromatin decompaction of chromatin that occurs during lymphocyte activation in response to phosphoinositol [33]. A few years later, Chi et al. performed a systematic study of the function of SMARCA4 at different stages of T lymphocyte differentiation and maturation into CD4+ and CD8+ populations. They found that Wnt and pre-T-cell-receptor signaling along with SMARCA4 are essential at successive stages of T-cell differentiation to change the chromatin landscape of the cells [31]. A conditional mutation in* Brg1* blocks T-cell differentiation [22, 31], and ablation of SMARCA4 results predominantly in a double negative (DN) (CD8− and CD4−) T-cell population. However, a small number of cells become CD4+ but fail to undergo maturation, resulting in a loss of T-cells [22, 31]. Mechanistically, SMARCA4 was demonstrated to bind to silencer elements and repress CD4 receptor expression in DN T-cells and to promote the activation of CD8 expression [34]. Interestingly, an N-terminal truncation of the HMG domain in SMARCE1 silences the CD4 receptor but not the expression of CD8, indicating that it functions in lineage choice [35]. In a recent study by Wan et al., the SMARCE1-containing SMARCA4 complex was shown to be required for remodeling chromatin at CD4 silencer regions, and this remodeling is crucial for the binding of the RUNX1 transcription repressor to the silencer DNA elements [36]. Holloway et al. provided direct evidence for chromatin remodeling by the BAF complex at the GM-CSF (granulocyte/macrophage colony stimulating factor) gene promoter in T-cells. They demonstrated a NF-*κ*B-dependent remodeling of a single nucleosome in the GM-CSF promoter upon T-cell activation [37].

Only a few studies have explored the role of BAF complexes in B-cell development. Mice deficient in* Baf155* and* Brg1* show defects in early B-cell development. Furthermore,* Baf155* deficiency results in low levels of B-cell-specific gene expression, loss of IL-7 signaling, lack of V(D)J rearrangements, and lack of activation of the TF EBF1 [38]. Our recent finding using CAGE expression profiling of epigenetic factors showed a B-cell-specific expression of DPF3 compared to hematopoietic progenitors and other mature cells, suggesting that this BAF subunit exhibits B-cell-specific function [28]. However, further studies are required to verify the biological function of DPF3 in B-cells.

Several TFs have been implicated in myeloid development and maturation. The CCAAT/enhancer binding protein (C/EBP) family of proteins participate in cell proliferation and terminal differentiation [39]. C/EBP and its TF isoforms are expressed at different stages of the myeloid branch [40]. One of the isoforms, C/EBP*β*, interacts with the BAF complex via its N-terminal activating domain to activate myeloid specific genes [41]. Vradii et al. further demonstrated that SMARCA4 is essential for the differentiation of myeloid progenitors to metamyelocytes when stimulated with G-CSF [23].

### 3.2. Imitation Switch (ISWI) Complexes

The ISWI subfamily of CRCs has a highly conserved ATPase catalytic core along with 1–3 auxiliary cofactors. In mammals, as many as seven different ISWI complexes have been described, including ACF (ATP-utilizing chromatin assembly and remodeling factor), CHRAC (chromatin remodeling and assembly complex), RSF (remodeling and spacing factor), WICH (WSTF-ISWI chromatin remodeling complex), NoRC (nucleolar remodeling complex), CERF (CECR2-containing remodeling factor), and NURF (nucleosome remodeling factor) [42, 43]. ACF, CHRAC, RSF, WICH, and NoRC contain SNF2H/SMARCA5, whereas CERF and NURF contain SNF2L/SMARCA1 as their catalytic SNF2 subunit. ISWI complexes are recruited to their nucleosomal substrates through their interactions with DNA-binding domains with the extranucleosomal DNA and/or by recognizing histone modifications to modulate chromatin structure [43, 44]. SMARCA5 is essential for early embryonic development and for hematopoietic progenitor cell proliferation. CD34+ progenitor cells lacking SMARCA5 are significantly stunted in their proliferative capacity and fail to respond to cytokines to induce differentiation [45].

Unlike the BAF complex, which is essential for T-cell development, the ISWI complex NURF is required for T-cell maturation only after the CD4+/CD8+ selection stage [46]. Landry et al. observed that mice carrying a conditional mutation of* Bptf*, the largest subunit of the NURF complex, displayed a significant reduction in single positive CD4+ and CD8+ T-cells. Furthermore, they observed that this defect in T-cell maturation was due to a change in the chromatin landscape as measured by DNase I hypersensitivity [46]. Thus, these studies indicate that different mammalian ISWI complexes are important for hematopoiesis and warrant further characterization to precisely understand the mechanistic details.

### 3.3. Chromodomain Helicase DNA Binding (CHD) Proteins

Mammals harbor nine different CHD proteins, which are characterized by the presence of tandem chromodomains and a SNF2 helicase domain. CHDs are further classified into three subfamilies depending on the presence of additional domains. CHD1-2 has an additional DNA-binding domain, CHD3–5 has a dual PHD domain, and CHD6–9 has a BRK domain. They are classified into subfamilies I, II, and III, respectively [47]. The signature motifs of CHDs, chromodomains, interact with DNA, RNA, and methylated histone tails, but these domains have variable affinities towards histone methylation [48, 49]. These enzymes act on chromatin substrates as a monomer in conjunction with other cofactors and with other remodeling complex [50, 51]. They have been implicated in diverse roles, such as orchestrating chromatin structure, transcription activation/repression, and histone turnover [52, 53]. CHD1 has been shown to be essential in maintaining open chromatin confirmation and the pluripotency of embryonic stem cells [54]. The yeast homolog of CHD1 is crucial for uniformly spacing nucleosomal arrays both* in vivo* and* in vitro* [55, 56]. Although much work has been performed, both biochemically and* in vivo* to study the biological role of CHD1, its function in hematopoiesis remains elusive. However, a study by Chen et al. hints at a potential role for CHD1 in bone marrow hematopoiesis and myeloid transformation through its function at Hox genes [57]. Nagarajan et al. showed that homozygous null* Chd2* mice have a defect in HSC differentiation into the erythroid lineage and that heterozygous mutant mice develop T-cell lymphomas [58].

The nucleosome remodeling and deacetylase (NuRD) complex consists of Mi-2*α*/CHD3 and Mi-2*β*/CHD4 as SNF2 ATPase subunits and two histone deacetylases, HDAC1 and HDAC2, and several auxiliary subunits [59]. The NuRD complex is diverse in its subunit composition, which dictates its specialized functions as a transcriptional corepressor. The variable subunits of the NuRD complex include the methyl-CpG binding domain proteins 2 and 3 (MBD2/3), the metastasis-associated proteins (MTA1/2/3), the retinoblastoma-associated binding proteins (RbAP46/48), a lysine-specific demethylase (LSD1/KRDM1), GATAD2A (p66*α*), and GATAD2B (p66*β*) [59, 60]. The Chd4/NuRD complex is important for both the self-renewal capacity of HSCs and lineage choice. HSCs deficient in Chd4 enter the cell cycle and differentiate into the erythroid lineage but not into other myeloid lineages or into lymphocytes [61], which exhausts HSCs and progenitors. Moreover, NuRD interacts with specific TFs, such as Ikaros and Helios, which are zinc-finger DNA-binding proteins that are critical for thymocyte development [62]. Furthermore, the Ikaros-NuRD interaction, which is primarily through CHD4 ATPase, is essential not only for thymocyte development but also for HSCs and B-cells [60]. This interaction guides the NuRD complex to specific gene targets through the DNA-binding activity of Ikaros [63].

The B-cells* Cd79a* gene encodes Ig*α*, which is essential for immunoglobulin assembly and intracellular signaling when the B-cell receptor interacts with an antigen. The activation of the gene encoding* Cd79a* is regulated not only by several TFs but also via different epigenetic mechanisms [64]. The CHD4 component of NuRD complex was found to be essential for repressing the transcription of Cd79*α*, as the knock-down of CHD4 increases transcription, accessibility of chromatin, and DNA demethylation [65]. Interestingly, the BAF complex activates the* Cd79a* promoter element in the presence of EBF and Pax5 TFs by binding to it, and the knock-down of SMARCA4/SMARCA2 reduces chromatin accessibility. The opposing function of NuRD and BAF complexes defines a delicate* Cd79a* expression balance [65]. Similar opposing functions for BAF and NuRD complexes have been observed for inflammatory response genes when macrophages were stimulated with lipopolysaccharide [66].

The differentiation and maturation of DN to DP T-cells are also regulated by the NuRD complex predominantly through its interaction with CHD4 ATPase. Conditional inactivation of CHD4 abrogates the expression of CD4 and reduces single positive CD4+ T-cells [67]. Furthermore, CHD4 binds to the enhancer element of CD4 and recruits histone acetyl-transferase p300 and the TF HEB, thereby supporting the transcription of CD4. Interestingly, Williams et al. did not find an association with HDAC2 in their coimmunoprecipitation assays, suggesting that CHD4 may act independently of the NuRD complex and may activate rather than repress genes [67]. In a separate study, the NuRD complex was shown to be associated with BCL11B (B-cell chronic lymphocytic leukemia/lymphoma 11B) through the MTA-1 subunit in CD4+ T-cells to promote transcriptional repression in target promoters [68].

### 3.4. Other CRCs

We also surveyed the existing evidence for roles of other SNF2 ATPases in hematopoiesis as follows. (1) The helicase lymphoid-specific enzyme (HELLS), also known as the lymphoid-specific helicase (LSH), is a member of the SNF2 helicase family. Loss of HELLS in mice results in a modest reduction in the transition of T-cells from the DN stage CD4−CD8− to the DP stage CD4+CD8+ [69]. (2) EP400 belongs to the SWR1 subfamily of SNF2 enzymes. An internal deletion within the catalytic core of EP400 yields defects in embryonic development and primitive hematopoiesis in mice [70]. EP400 conditional knockout mice exhibit embryonic lethality within two weeks, with acute loss of bone marrow HSCs and impaired cell cycle progression [71].

## 4. SNF2 Expression Patterns in Human Blood Cells

Genome-wide methodologies to study gene expression provide a holistic picture of promoter usage, levels of mRNA, and proteins within cells/tissues. To construct a comprehensive picture of the levels of expression of genes encoding epigenetic factors in various blood cells, we exploited the Cap Analysis of Gene Expression (CAGE) technique as a part of the Fantom 5 collaborative project (http://fantom.gsc.riken.jp/) [28]. To better assess the gene expression levels, we analyzed the mRNA expression of genes encoding SNF2 ATPases separately from the genes encoding the CRC auxiliary subunits (Figures 2(a) and 2(b)). Interestingly, we observed that several of these genes were expressed at different levels. Additionally, some genes were abundantly expressed, including genes encoding SNF2 ATPases and CRC auxiliary subunits such as CHD2, CHD4, BTAF1, SMARCA5 and BAZ1A, EPC1, and MORF4L2, respectively (Figures 2(a) and 2(b)). However, CHD5 and SMARCA1 were not expressed in any of the blood cells tested (Figure 2(a)) consistent with the fact that they are highly expressed in neuronal tissue [72, 73]. This suggests a broad function for some of the SNF2 genes in hematopoietic cells physiology. Moreover, we found progenitor-specific expression for some genes, such as HELLS and HLTF, suggesting that these factors may play important roles in maintaining HSCs (Figure 2(a)). Furthermore, some genes had lineage-specific expression patterns; for example, CHD3 displayed higher transcript levels in the lymphoid lineage compared with the myeloid lineage [28]. Thus, genome-wide CAGE transcriptome profiling in hematopoietic cells has given overview of expression patterns of not only SNF2 ATPases but also auxiliary subunits, important to understand combinatorial assembly of CRCs (discussed in the following section).

We also included several leukemic cell lines in our epigenetic transcriptome analysis and found that the leukemic cell lines clustered together with progenitor cells based on their expression profiles [28]. This finding suggests that they probably share epigenetic mechanisms required for self-renewal. Conversely, we also observed significant differences in the expression levels of several genes encoding SNF2 enzymes and CRC subunits compared with normal hematopoietic cells, suggesting a putative role in promoting the leukemic phenotype. Systematic studies of both normal hematopoietic cells and leukemic cell lines provide a broader perspective of SNF2 family enzymes expression; however, detailed investigations are required to understand their molecular functions in normal and malignant hematopoiesis.

## 5. CRC Isoforms in Hematopoiesis

CRCs are often multisubunit complexes containing one or two SNF2 ATPase subunits that are capable of hydrolyzing ATP along with several auxiliary subunits. Subsequently, they can convert energy released from ATP hydrolysis to mechanical energy for changing chromatin confirmations. However, such mechanical actions are not only performed by the ATPase subunit but also require the participation of the entire complex [44]. Although auxiliary subunits do not individually catalyze ATP hydrolysis or chromatin remodeling, they do dictate the specific functions of a CRC [53]. Both biochemical and* in vivo* functional data are now available, which explains the specific roles of different CRC subunits in programming the development and differentiation of cells [18, 44]. This mechanism of action was recently highlighted in neuronal development [74], heart and muscle development [75, 76], and in controlling the development of blood cell lineages [13, 20]. In our recent CAGE expression study, we identified several potential auxiliary subunits that were either hematopoietic cell- or lineage-specific. For example, BAF45C/DPF3, BAF53A/ACTL6A, and BAF60C/SMARCD3 transcripts were enriched in B-cells, hematopoietic progenitor, and monocytic cells, respectively (Figure 2(b)) [28]. Previously, Lickert et al. showed that tissue-specific SMARCD3 containing BAF complex is essential in heart morphogenesis and knockdown of SMARCD3 results in defect in heart development [76]. Similarly DPF3-BAF complex is essential for both heart and muscle development [75]. Recently, Krasteva et al. highlighted the role of ACTL6A-BAF complex in proliferation of HSCs and adult hematopoiesis [20]. Much work is required to investigate the combinatorial association of auxiliary subunits of SNF2 ATPases in hematopoiesis.

The ATPase motors in the BAF complex with either SMARCA4/Brg1 or SMARCA2/Brm share significant identity in their amino acid composition and biochemical chromatin remodeling activities. However, notably, SMARCA4 is essential, whereas SMARCA2 is dispensable [30]. Such distinct biological activities have been attributed to the nonconserved N-terminal regions of the ATPases, which are potential targets for different TFs and signaling molecules and therefore regulate distinct cellular processes [77]. More recently, the PBAF complex with SMARCA4 as the catalytic core was demonstrated to interact with CHD7, to cooccupy the distal enhancer element of Sox9 and activate neural crest formation [51]. Phelan et al. demonstrated that SMARCA4, BAF45, BAF155/SMARCC1, and BAF170/SMARCC2 subunits can form a functional SMARCA4 complex that is capable of remodeling nucleosomes [78]. What, then, is the function of the other subunits associated with the BAF complex? The answer lies in understanding the diverse and dynamic functions of the BAF complex through its association with specific auxiliary cofactors in cell/tissue-specific manner [18]. The underlying mechanism for the existence of diverse BAF complexes lies in the presence of different domains within specific auxiliary subunits, which can read posttranslational histone modification in context of chromatin in a tissue specific manner. For example, DPF3 contains two PHD (plant homeodomain) fingers which recognize both methylated and acetylated histone residues and direct BAF complex to specific genomic targets [75]. We cannot rule out the existence of similar mechanisms in hematopoietic development, which thus warrants further investigation. These studies thus reflect the dynamic mechanisms of action of BAF complexes at different levels in promoting normal tissue development.

CRCs can also form subcomplexes with transcription factors to target specific genes. For example, the transcription factor EKLF (erythroid Krüppel-like factor) binds to the *β*-globin gene promoter and activates its transcription [79]. Biochemical characterization revealed interactions between EKLF and SMARCA4, SMARCC1, SMARCC2, BAF47/SMARCB1/hSNF5, and BAF57/SMARCE1 in a subcomplex called E-RC1 (EKLF coactivator remodeling complex 1). This complex can remodel chromatin and facilitate the transcription of chromatinized templates of the *β*-globin gene promoter* in vitro* [21]. Interestingly, the BAF complex cannot substitute for E-RC1 in *β*-globin gene expression, indicating that E-RC1 has a specialized function [80].

The composition of the NuRD complex is dynamic and changes with its distinct roles in cell survival, differentiation, development, and homeostasis in various organisms [81]. For example, the MTA3-NuRD complex is essential for primitive hematopoiesis in the Zebrafish [82] and for B-cell-specific transcriptional programming with the Bcl6 transcriptional repressor [83]. MBD2 can replace MBD3 in NuRD to form a distinct complex with nonoverlapping functions [84]. Intriguingly, MBD3-deficient mice are embryonic lethal, whereas MBD2 deficient mice develop normally [85]. Further studies by Kaji et al. showed that the MBD3-NuRD complex is essential for the pluripotency of embryonic stem cells [86]. An LSD1 containing NuRD complex was identified in breast cancer cells, where it demethylates histone H3 at amino acids lysine 4 and lysine 9 [87]. Although LSD1 participates in several stages of HSC maintenance and differentiation, its function in complex with NuRD in hematopoiesis remains elusive [88].

Thus, we can conclude that CRC composition is diverse in blood cells and that several different CRC isoforms exist for specialized function.

## 6. Aberrant Expression of SNF2 ATPases and CRC Auxiliary Subunits in Leukemia

Dysfunctional expression of CRC subunits has been associated with leukemia. Downregulation of the BAF complex subunits SMARCA4, ARID1A, and SMARCB1 has been associated with glucocorticoid-resistance in acute lymphoid leukemia (ALL) [89].

**Figure 3:**
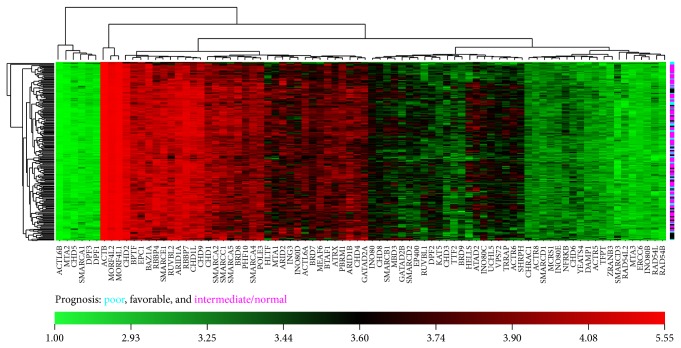
Expression of CRCs in subtypes of AML patients. The gene expression data from 194 AML patients were mined for CRC subunits from the TCGA (The Cancer Genome Atlas) data portal (https://tcga-data.nci.nih.gov/tcga/). The expression values were log10 transformed, and hierarchal clustering and the heatmap were constructed using CIMminer. The colored bar on the right side of the heatmap indicates patients with a poor (blue), favorable (black), and intermediate/normal (pink) prognosis.

Acute myeloid leukemia (AML) is a heterogeneous disease that consists of several subtypes based on cytogenetic and genetic alterations, which form the basis for classification and prognosis. AML is characterized by disturbed transcriptional regulation, which leads to a differentiation block and increased proliferation. Several chromosomal translocations or mutations in AML involve epigenetic regulators (e.g., DNMT3a, MLL, and TET2) or transcription factors (e.g., C/EBP*α* and AML1) (reviewed in [90]). Transcription factors that regulate hematopoiesis are also targets for the aberrant epigenetic regulation that modulate their expression levels, which affect cell differentiation. For example, patients with a mutated DNMT3A gene have genomes enriched for DNA demethylation at genes that encode for TFs [91]. Although the expression patterns of CRCs are, in most cases, similar to the expression patterns of normal myeloid progenitor cells and are not deregulated in AML, some genes encoding for SNF2 enzymes and CRC subunits are aberrantly expressed in AML compared with their normal counterparts (Table 1). Our analysis of SNF2 family enzymes and CRC auxiliary subunits in different AML subtypes using the AML cohort from The Cancer Genome Atlas (TCGA) revealed that the expression patterns are not sufficiently specific to cluster the patients based on prognosis [92] (Figure 3). However, some of the genes display large variations in expression between patients (Figure 3). For example, the expression levels for HELLS, RUVBL1, and EP400 differ by 17-, 12-, and 5-fold, respectively, between the three patients with the highest or lowest expression. RUVBL1 is located on a chromosomal region that is frequently rearranged in leukemia and solid tumors, which further suggests a role for RUVBL1 in leukemogenesis [93, 94]. The AML blast cells highly express SMARCA5, and its expression goes down upon treatment [95]. We observed high levels of SMARCA5 transcripts from the patient data extracted from TCGA (Figure 3). This may contribute to increased proliferation of leukemic cells because SMARCA5 has a role in promoting the proliferation of hematopoietic progenitor cells (as mentioned above). CTCF and PU.1 are key TFs that regulate the growth and differentiation of myeloid cells [96, 97]. CTCF and SMARCA5 cooperatively interact with the DNA regulatory elements of PU.1. Consequently, the SMARCA5 interaction increases the expression of PU.1, which supports myeloid differentiation. Interestingly, the success of this interaction depends on the DNA methylation status of the PU.1 regulatory elements. Azacytidine treatment of AML blast cells demethylates DNA and restores PU.1 expression, thereby enabling myeloid differentiation [98]. These studies reflect upon the mode of action of SMARCA5 and regulation of PU.1 expression in patients suffering from AML.

A perturbed regulatory balance can contribute to carcinogenesis depending on the cellular context. The SNF2 ATPase CHD1 has a dual role in cancer. The CHD1 gene is overexpressed in AML cells, possibly indicating an oncogenic role in leukemia (Table 1). However, prostate cancer patients frequently harbor Chd1 gene deletions, indicating that Chd1 may act as a tumor suppressor in the prostate [99, 100]. The downregulation of HELLS in AML patients (Table 1) is interesting because HELLS has been demonstrated to have a role in DNA repair. The phosphorylation of H2AX at double strand breaks is dependent on the ability of HELLS to hydrolyze ATP and is required for successful DNA repair [101]. Deregulated DNA methylation is a hallmark of AML, and HELLS represses transcription via interacting with the DNA methyltransferase DNMT1 [102, 103]. HELLS regulates HOX gene expression during normal development [104]. The downregulation of HELLS in AML may dysregulate the expression of HOX genes that is associated with both AML and ALL (reviewed in [105]). AML cells display a shift in their SWI/SNF complex subunits, with ARID1A being overexpressed while the expression of ARID1B is reduced compared with normal counterparts (Table 1). The role of ARID1A in adult hematopoiesis is not clear, but ARID1A activity in stroma cells has been demonstrated to control the size of the fetal HSC pool [106]. The role of ARID1B in hematopoiesis and leukemia is less clear, but loss of function of ARID1B has been proposed to have tumor suppressor function in lung and pancreatic cancer [107, 108]. The downregulation in AML suggests a similar tumor suppressor function for ARID1B in blood. The expression of another SWI/SNF complex subunit, ACTL6A, is also repressed in AML. ACTL6A has been shown to be essential for adult hematopoiesis and HSC maintenance [20]. However, its role in AML remains to be elucidated.

The BAF complex has been suggested to have different compositions in HSC and leukemic cells [109]. The BAF complex in leukemic cells (leukBAF complex) contains the catalytic subunit SMARCA4, which is dispensable for maintenance of LT-HSC but is required for proliferation and multipotency [109]. Leukemic cells lacking SMARCA4 undergo cell cycle arrest and apoptosis. The expression pattern of catalytic and complex subunits in long-term quiescence HSC (LT-HSC) suggest that LT-HSC has a BAF complex containing SMARCA2 as the catalytic subunit dominant. Accordingly, Buscarlet et al. proposed that the BAF complex composition switch between HSC and leukemic cells contributes to leukemogenesis [109]. Mechanistically, Shi et al. showed that SMARCA4 interacts with Myc enhancer elements, remodel chromatin to promote TF occupancy and long range interaction with the Myc promoter to maintain leukemogenesis [110]. Such mechanisms give insight as to how leukemic cells manipulate the function of CRCs to maintain cancerous nature.

## 7. SNF2 and CRC Mutations in Leukemia

In recent years, large-scale genomics efforts based on new parallel sequencing techniques, such as the International Cancer Genome Consortium (ICGC) and the Cancer Genome Atlas (TCGA), have provided large datasets that enable systematic studies of mutations in epigenetic machinery in cancer. Genes encoding enzymes involved in basic epigenetic mechanisms, such as histone modification, chromatin remodeling, and DNA methylation, are frequently mutated in cancer, including a range of tissue types such as breast, brain, lung, ovarian, blood, kidney, colon, uterus, liver, and pancreas (reviewed in [90]). Mutations in genes encoding SNF2 enzymes and CRC subunits have been identified in different blood cancers. For example, the gene for HELLS is frequently truncated by 75 base pairs in AML (56.7%) and ALL (37%) patients [111]. Another example is a mutation in the SMARCB1 subunit of BAF complex, which was reported to cause a loss of function in 24% of chronic myeloid leukemia patients [112]. Here, we focus on the occurrence of mutations of chromatin remodelers in blood cancers. The ICGC data portal was queried with all 30 human SNF2 helicase genes and 67 genes encoding CRC auxiliary subunits (October 2014). Frequent mutations in SNF2 genes were found in malignant lymphoma patients in a German cohort of 44 patients and in a cohort 75 of acute myeloid leukemia (AML) patients from South Korea. The SNF2 genes SMARCA4 (34%), ZRANB3 (32%), and CHD9 (20%) were the three most frequently mutated genes in patients with malignant lymphoma, whereas CHD3 was the most frequently mutated gene in AML (Table 2). There are also relatively frequent mutations in genes encoding some SNF2 enzymes, that is, the CHD2 enzyme (4.6%) in a Spanish cohort of 109 Chronic Lymphocytic Lymphoma (CLL) patients. Thus, mutations in SNF2 genes appear to be quite common in blood cancers.

SMARCA4 is one of the catalytic subunits of the BAF complex. Subunits of BAF complex are mutated in a variety of cancers: loss of function of the auxiliary subunit SMARCB1 was detected in 98% of rhabdoid tumors and in 30–40% of familial Schwannomatosis; the subunit PBRM1 was mutated in 41% of renal carcinoma; the subunit ARID1A was mutated in 50% of ovarian clear cell carcinoma and in 35% of endometrial carcinoma; and SMARCA4 itself was mutated in 35% of nonsmall cell lung cancers [113]. We used the ICGC data portal to assess whether these and other CRC subunits are mutated in hematological malignancies. We observed that mutations were frequent in malignant lymphoma (Germinal center B-cell derived lymphomas) for the BAF ATPase SMARCA2 (18%) and auxiliary subunits ARID1A (18%), ARIDB (32%), ARID2 (18%), and DPF3 (23%), and other subunits were also mutated at lower frequencies (Table 3). These frequencies are higher than a report of mutations in a cohort of 68 Diffuse large B-cell lymphoma patients, who harbored 16.2% of mutations in any gene encoding BAF subunits and 2.9% for ARID1A and 5.9% for ARID1B [114]. Interestingly, the ICGC portal showed that some BAF subunits were frequently mutated in the South Korean cohort of AML patients, that is, PBRM1 (6.7%), SMARCC1 (6.7%), and DPF3 (6.7%). However, significantly lower frequencies of SWI/SNF mutations were identified in the Spanish CLL cohort (4 mutations in 109 patients), which agrees with the study by Shain and Pollack [114], who identified 8 mutations in any BAF subunit gene in a cohort of 196 CLL patients.

The subunits of the other CRC complexes were also mutated in patients with lymphoma, though at frequencies somewhat lower compared with SWI/SNF. For example, mutations in the NuRD complex subunits MTA3 (18%) and p66beta/GATAD28 (16%) were observed. The MRG15 subunit of the EP400 complex was mutated in 16% of patients with malignant lymphoma and in 5.3% of AML patients. CRC mutations were rare in the CLL cohort. Thus, in agreement with the central role for SNF2 enzymes and their CRC complexes in blood cell differentiation, mutations are often observed in patients with malignant lymphoma and AML, suggesting that disruptions in the function of these enzymes and complexes could contribute to these blood cancer phenotypes.

## 8. Conclusions

SNF2 family enzymes are key players in the epigenetic control of cell/tissue development, differentiation, and maturation. The question is how and by what mechanisms SNF2 enzymes and CRCs regulate epigenetic switches? Decades of research by several groups have shown that these enzymes change the accessibility of DNA by multiple chromatin remodeling mechanisms. Such mechanisms are critical for maintenance of genome integrity, regulation of transcription, and organization of chromatin into higher-order structures. Biochemical experiments to assess the functions of CRCs in a homogeneous* in vitro* assembled chromatin system revealed that all CRCs tested so far translocate on the nucleosomal DNA [7]. This DNA translocase activity results in different chromatin remodeling outcomes. For example, yeast SWI/SNF can slide as well as evict nucleosomes while ISW2 mostly slides nucleosomes [7, 43, 44]. This shows that different SNF2 family enzymes remodel chromatin by both redundant and nonredundant mechanisms. However, access to the target chromatic loci and remodeling by CRCs* in vivo* are more complex. Since CRCs lack motifs to recognize specific DNA sequences, other factors are required to recruit CRCs to their genomic targets. The interactions of CRCs with transcription factors and posttranslational histone modifications perform this function in cell/tissue specific manner.

Recent studies have shown that the auxiliary subunit composition of CRCs is dynamic and variable during cellular development. Such diversification adds to another level of complexity to the current understanding of gene regulation by CRCs in heart, muscle, and neuronal development as discussed above. More recently, studies have shown that the multilineage hematopoietic system possesses a similar mode of regulation where subunit composition of CRCs such as BAF and NuRD changes during hematopoietic lineage choices. Our genome-wide expression data revealed several such differentially expressed CRC components [28]. In an elegant study by the Lessard group the necessity of ACTL6A for maintaining the HSC pool and that the SMARCA4 containing BAF to be critical for promoting leukemogenesis was demonstrated [20, 109]. The Vakoc group recently provided insights into the mechanism of leukemogenesis where the SMARCA4 containing BAF complex interacts with the Myc enhancer element and via chromatin looping to the Myc promoter to stimulate its oncogenic transcription [110]. In heart and muscle development the key auxiliary CRC subunit DPF3 targets chromatin through its PHD domains, which interact with acetylated and methylated chromatin regions [75, 76]. We show that DPF3 is specifically expressed in B lymphocytes; however its biological relevance in blood cells still needs to be established [28].

Defects in chromatin remodeling caused by mutations in the genes encoding SNF2 ATPases and CRC auxiliary subunits are common in leukemia. Data mining from ICGC consortium shows that these mutations are common in genes encoding SNF2 family enzymes. We also observed aberrant expression of genes encoding SNF2 family enzymes and CRC subunits in acute myeloid leukemia (AML) using data from the Cancer Genome Atlas (TCGA). Mechanistic studies will clearly be important to understand the contribution of these mutations and expression changes to leukemogenesis and possibly to allow for the development of personalized therapies based on specific SNF2 or CRC targets. In this review, we have attempted to summarize the current knowledge of SNF2 and CRC functions in hematopoiesis and leukemia. Further studies are required to unfold multiple mechanisms of chromatin remodeling complexes by which hematopoietic cells can follow normal or malignant hematopoiesis.

## Figures and Tables

**Table 1:** Aberrant expression of SNF2 enzymes and CRC subunits in AML.

	Upregulated in AML	Downregulated in AML
SNF2 enzymes	CHD1 FC 1.9	
	CHD2 FC 4.9	
		HELLS FC 0.69
		SMARCAD1/Etl1 FC 0.48
		TTF2 FC 0.54
		HLTF FC 0.6

SWI/SNF complex subunits	ARID1A/BAF250a FC 2.1	
	SMARCD1/BAF60A FC 1.7	
	SMARCC2/BAF170 FC 2.5	
		ARID1B/BAF250b FC 0.6
		ACTL6A/BAF53A FC 0.4
		SMARCE1/BAF57 FC 0.7

INO80 complex	INO80D/FLJ20309 FC 1.5	

The table shows CRC with significantly changed expression (*P* < 0.0001) in AML compared to its closest normal counterpart at Hema Explorer [115]. The AML cohort consist of 144 patients with the following subtypes: 39 patients with t(8:21), 38 patients with t(15:17), 29 patients with inv(16)/t(16;16), and 38 patients with t(11q23)/MLL. FC = fold change.

**Table 2:** ICGC data for the SNF2 enzymes.

SNF2 enzyme	Mutations in malignant lymphoma	Mutations in AML	Mutations in CLL
SMARCA4/Brg1	15/44 (34%)	2/75 (2.7%)	—
SMARCA2/Brm	8/44 (18%)	3/75 (4.0%)	1/109 (0.92%)
SMARCA5/SNF2H	1/44 (2.3%)	—	—
SMARCA1/SNF2L	5/44 (11%)	—	—
CHD1	2/44 (4.5%)	2/75 (2.7%)	—
CHD2	5/44 (11%)	4/75 (5.3%)	5/109 (4.6%)
CHD3	—	4/75 (5.3%)	—
CHD4	5/44 (11%)	3/75 (4.0%)	—
CHD5	1/44 (2.3%)	3/75 (4.0%)	—
CHD6	6/44 (14%)	4/75 (5.3%)	—
CHD7	8/44 (18%)	1/75 (1.3%)	—
CHD8	3/44 (6.8%)	3/75 (4.0%)	—
CHD9	9/44 (20%)	3/75 (4.0%)	—
HELLS	4/44 (9.1%)	3/75 (4.0%)	—
CHD1L	3/44 (6.8%)	1/75 (1.3%)	—
SRCAP	2/44 (4.5%)	1/75 (1.3%)	
EP400	3/44 (6.8%)	2/75 (2.7%)	—
INO80	7/44 (16%)	1/75 (1.3%)	—
SMARCAD1/Etl1	3/44 (6.8%)	1/75 (1.3%)	
RAD54B	2/44 (4.5%)	3/75 (4.0%)	—
RAD54L	4/44 (9.1%)	1/75 (1.3%)	—
ATRX	—	2/75 (2.7%)	—
Arip4	ND	ND	ND
SMARCA3	3/44 (6.8%)	2/75 (2.7%)	—
TTF2	2/44 (4.5%)	1/75 (1.2%)	—
SHPRH	8/44 (18%)	3/75 (4.0%)	1/109 (0.92%)
BTAF1	7/44 (16%)	2/75 (2.7%)	—
ERCC6	2/44 (4.5%)	2/75 (2.7%)	—
SMARCAL1	2/44 (4.5%)	—	—
ZRANB3	14/44 (32%)	3/75 (4.0%)	—

The ICGC data portal (https://dcc.icgc.org/) was queried with all 30 human SNF2 helicase genes (October 2014). The frequency and percent of mutations in the patients are indicated for each gene. ND = not determined.

**Table 3:** ICGC data for the noncatalytic CRC subunits.

CRC complex subunits	Mutations in malignant lymphoma	Mutations in AML	Mutations in CLL
SWI/SNF complex:			
SMARCA4/Brg1	15/44 (34%)	2/75 (2.7%)	—
SMARCA2/Brm	8/44 (18%)	3/75 (4.0%)	1/109 (0.92%)
ARID1A/BAF250a	8/44 (18%)	2/75 (2.7%)	2/109 (1.8%)
ARID1B/BAF250b	14/44 (32%)	1/75 (1.3%)	—
ARID2/BAF200	8/44 (18%)	—	—
BAF180/PBRM1	—	5/75 (6.7%)	—
SMARCC1/BAF155	5/44 (11%)	5/75 (6.7%)	—
SMARCC2/BAF170	—	—	—
SMARCD1/BAF60A	—	—	—
SMARCD2/BAF60B	2/44 (4.5%)	1/75 (1.3%)	—
SMARCD3/BAF60C	4/44 (9.1%)	—	—
ACTL6A/BAF53A^*^	5/44 (11%)	—	—
ACTL6B/BAF53B	1/44 (2.3%)	—	—
SMARCB1/BAF47/hSNF5	2/44 (4.5%)	2/75 (2.7%)	—
BAF45A/PHF10	3/44 (6.8%)	—	—
DPF1/BAF45B	—	1/75 (1.3%)	—
DPF3/BAF45C	10/44 (23%)	5/75 (6.7%)	—
DPF2/BAF45D	—	1/75 (1.3%)	1/109 (0.92%)
SMARCE1/BAF57	3/44 (6.8%)	—	—
BRD7	2/44 (4.5%)	—	—
BRD9	—	1/75 (1.3%)	—
*β*-actin/ACTB	5/44 (11%)	—	—
INO80 complex:			
INO80	7/44 (16%)	1/75 (1.3%)	—
*β*-actin/ACTB^*^	5/44 (11%)	—	—
ACTL6A/BAF53A^*^	1/44 (2.3%)	—	—
Arp5/ACTR5	1/44 (2.3%)	1/75 (1.3%)	—
Arp8/ACTR8	1/44 (2.3%)	3/75 (4.0%)	—
RUVBL1/TIP49A^*^	3/44 (6.8%)	2/75 (2.7%)	—
RUVBL2/TIP49B^*^	3/44 (6.8%)	2/75 (2.7%)	—
IES2/INO80B/PAPA-1	1/44 (2.3%)	2/75 (2.7%)	1/109 (0.92%)
IES6/INO80C/c18orf37	3/44 (6.8%)	3/75 (4.0%)	—
YY1	4/44 (9.1%)	—	—
UCH37/UCHL5	3/44 (6.8%)	—	—
NFRKB/INO80G	—	3/75 (4.0%)	—
MCRS1/MCRS2/MSP58/INO80Q	2/44 (4.5%)	—	—
TFPT/Amida/INO80F	ND	ND	ND
INO80D/FLJ20309	4/44 (9.1%)	1/75 (1.3%)	—
INO80E/CCDC95/FLJ90652	2/44 (4.5%)	1/75 (1.3%)	—
EP400 complex: See shared subunits^*^ and additional subunits below			
EP400	3/44 (6.8%)	2/75 (2.7%)	—
BRD8	2/44 (4.5%)	2/75 (2.7%)	—
TRRAP	6/44 (14%)	3/75 (4.0%)	—
Tip60/KAT5	2/44 (4.5%)	—	—
MRG15	7/44 (16%)	4/75 (5.3%)	—
MRGX	—	2/75 (2.7%)	—
FLJ11730	1/44 (2.3%)	1/75 (1.3%)	—
MRGBP	2/44 (4.5%)	2/75 (2.7%)	—
EPC1	6/44 (14%)	1/75 (1.3%)	—
ING3	3/44 (6.8%)	1/75 (1.3%)	—
SRCAP complex:			
SRCAP	2/44 (4.5%)	1/75 (1.3%)	—
RUVBL1/TIP49A^*^	3/44 (6.8%)	2/75 (2.7%)	—
RUVBL2/TIP49B^*^	3/44 (6.8%)	2/75 (2.7%)	
ACTL6A/BAF53A^*^	5/44 (11%)	—	—
ACTR6	ND	ND	ND
GAS41^*^	2/44 (4.5%)	1/75 (1.3%)	—
DMAP1^*^	1/44 (2.3%)	2/75 (2.7%)	—
YL-1^*^	2/44 (4.5%)	1/75 (1.3%)	—
NuRD complex (Mi-2):			
CHD4	5/44 (11%)	3/75 (4.0%)	—
CHD3	—	4/75 (5.3%)	—
MBD3	—	—	—
MTA1	3/44 (6.8%)	2/75 (2.7%)	1/109 (0.92%)
MTA2	1/44 (2.3%)	1/75 (1.3%)	
MTA3	8/44 (18%)	—	—
HDAC1	3/44 (6.8%)	—	—
HDAC2	4/44 (9.1%)	3/75 (4.0%)	—
RBBP7/RbAp46	1/44 (2.3%)	1/75 (1.3%)	—
RBBP4/RbAp48	—	2/75 (2.7%)	—
p66-*α*/GATAD2A	5/44 (11%)	1/75 (1.3%)	—
p66-*β*/GATAD2B	7/44 (16%)	1/75 (1.3%)	—
NURF complex /SMARCA1 (SNF2L)	5/44 (11%)	—	—
BPTF	3/44 (6.8%)	1/75 (1.3%)	—
RBBP7/RbAp46	1/44 (2.3%)	1/75 (1.3%)	—
RBBP4/RbAp48	—	2/75 (2.7%)	—
CHRAC complex SMARCA5 (SNF2H)	1/44 (2.3%)	—	—
hACF1/WCRF180	4/44 (9.1%)	—	—
hCHRAC17/POLE3	1/44 (2.3%)	—	—
hCHRAC 15	—	—	—

The ICGC data portal (https://dcc.icgc.org/) was queried with 67 genes encoding CRC subunits (October 2014). The frequency and percentage of mutations in the patients are indicated for each gene. Shared CRC subunits are marked with an asterisk (∗). ND = not determined.
